# Correlates of and Body Composition Measures Associated with Metabolically Healthy Obesity Phenotype in Hispanic/Latino Women and Men: The Hispanic Community Health Study/Study of Latinos (HCHS/SOL)

**DOI:** 10.1155/2019/1251456

**Published:** 2019-01-15

**Authors:** Mayra L. Estrella, Amber Pirzada, Ramon A. Durazo-Arvizu, Jianwen Cai, Aida L. Giachello, Rebeca Espinoza Gacinto, Anna Maria Siega-Riz, Martha L. Daviglus

**Affiliations:** ^1^Institute for Minority Health Research, University of Illinois at Chicago, 1819 West Polk Street, Chicago, IL 60612, USA; ^2^Division of Biostatistics, Public Health Sciences, Loyola University Chicago, 2160 South First Avenue, Maywood, IL 60153, USA; ^3^Collaborative Studies Coordinating Center, Department of Biostatistics, University of North Carolina at Chapel Hill, 123 W. Franklin Street, Chapel Hill, NC 27516, USA; ^4^Department of Preventive Medicine, Northwestern University, 680 N Lake Shore Dr Suite, Chicago, IL 60611, USA; ^5^Graduate School of Public Health, San Diego State University, 5500 Campanile Drive, San Diego, CA 92182, USA; ^6^School of Nursing and Departments of Public Health Sciences and Obstetrics and Gynecology, School of Medicine, University of Virginia, P.O. Box 800717, Charlottesville, VA 22908, USA

## Abstract

**Background:**

Individuals with “metabolically healthy obesity” (MHO) phenotype (i.e., obesity and absence of cardiometabolic abnormalities: favorable levels of blood pressure, lipids, and glucose) experience lower risk of cardiovascular disease compared with those with “metabolically at-risk obesity” (MAO) phenotype (i.e., obesity with concurrent cardiometabolic abnormalities). Among Hispanic/Latino women and men with obesity, limited data exist on the correlates of and body composition measures associated with obesity phenotypes.

**Methods:**

Data from the Hispanic Community Health Study/Study of Latinos (2008–2011) were used to estimate the age-adjusted distribution of obesity phenotypes among 5,426 women and men (aged 20–74 years) with obesity (BMI ≥ 30 kg/m^2^) and to compare characteristics between individuals with MHO and MAO phenotypes. Weighted Poisson regression models were used to examine cross-sectional associations between 1-standard deviation (SD) increase in body composition measures (i.e., body fat percentage, waist circumference, and body lean mass) and MHO phenotype prevalence.

**Results:**

The age-adjusted proportion of the MHO phenotype was low (i.e., 12.5% in women and 6.5% in men). In bivariate analyses, women and men with the MHO phenotype were more likely to be younger, have higher education and acculturation levels, report lower lifetime cigarette use, and have fasting insulin and waist circumference levels than MAO. Adjusting for sociodemographic and lifestyle factors, among women, each 1-SD increase in body fat percentage, waist circumference, and lean body mass was, respectively, associated with a 21%, 33%, and 31% lower prevalence of the MHO phenotype. Among men, each 1-SD increase in waist circumference and lean body mass was, respectively, associated with a 20% and 15% lower prevalence of the MHO phenotype.

**Conclusions:**

We demonstrated that higher waist circumference and higher lean body mass were independently associated with a lower proportion of the MHO phenotype in Hispanic/Latino women and men. Findings support the need for weight reduction interventions to manage cardiometabolic health among Hispanics/Latinos.

## 1. Introduction

Obesity (defined as body mass index (BMI) ≥30 kg/m^2^) is associated with increased risk of cardiovascular disease (CVD) [1] and mortality [2], higher health-care costs [3], and lower quality of life [4]. Individuals with obesity have high rates of concurrent cardiometabolic conditions such as hypertension, hyperlipidemia, and insulin resistance [5, 6]. However, a subset of individuals with obesity are free of such cardiometabolic conditions, that is, have the “metabolically healthy obesity” (MHO) phenotype [7, 8]. The MHO phenotype has been associated with lower risk of CVD compared with the “metabolically at-risk obesity” (MAO) phenotype (i.e., obesity with concurrent unfavorable blood pressure, lipid, and glucose profiles) [9], although not all evidence suggests a protective effect [10, 11].

Previous studies among primarily non-Hispanic white and international populations have tried to identify factors that may distinguish the MHO from the more common MAO phenotype [12–17]. Most of these studies have focused on lifestyle or behavioral factors as determinants that may differentiate obesity phenotypes; some studies have reported associations of better diet quality and higher physical activity levels with the MHO phenotype [12, 15–17], but other studies have reported null associations [14, 15]. Furthermore, little is known about the association between body composition measures (i.e., body fat percentage, waist circumference, and lean body mass) and obesity phenotypes. A study found that there were no associations of body fat percentage and lean body mass with the MHO phenotype in non-Hispanic white women [18]. Other studies have documented that higher waist circumference is associated with a lower prevalence of the MHO phenotype in adults from diverse backgrounds [11, 12].

In the United States (US), the Hispanic/Latino population compared with non-Hispanic whites is disproportionally affected by the obesity epidemic [19, 20] and experience a high burden of CVD risk factors [21]. However, there are limited national data on the proportion and correlates of metabolic phenotypes among diverse US Hispanic/Latino women and men with obesity. A better understanding of the association between body composition measures and obesity phenotypes is warranted because the behavioral and clinical determinants of cardiometabolic health among individuals with obesity remain largely unknown. Therefore, among US Hispanic/Latino women and men with obesity, the purpose of this study was to describe the behavioral and clinical correlates of the MHO phenotype and to examine the cross-sectional associations between body composition measures and the MHO phenotype using data from the Hispanic Community Health Study/Study of Latinos (HCHS/SOL). It is hypothesized that those with the MHO phenotype have more favorable profiles of behavioral and clinical factors compared with those with the MAO phenotype. We also hypothesize that the higher body fat percentage and higher waist circumference will be independently associated with a lower prevalence of the MHO phenotype but that higher lean muscle mass will be associated with a higher prevalence of the MHO phenotype, regardless of sociodemographic and behavioral factors.

## 2. Materials and Methods

### 2.1. Study Design and Analytic Sample

The HCHS/SOL is a multicenter population-based study designed to examine CVD risk factors in Hispanic/Latino adults of diverse backgrounds (i.e., Cuban, Central American, Dominican, Mexican, Puerto Rican, and South American). Details of the HCHS/SOL sampling design, cohort selection, and study protocols have been previously reported [22, 23]. Briefly, a stratified two-stage sampling design was used to recruit self-identified Hispanics/Latinos (*N* = 16,415) aged 18–74 years at baseline (2008–2011). Study enrollment was conducted from households located in four US metropolitan areas: Bronx, NY; Chicago, IL; Miami, FL; and San Diego, CA. Institutional review boards of affiliated sites approved the study, and participants provided written informed consent.

Among the 16,415 participants enrolled in the study, there were 6,978 participants with obesity (i.e., BMI ≥ 30 kg/m^2^). Of those 6,978, we excluded 136 participants aged <20 years to facilitate comparisons with national estimates of obesity phenotypes in US Mexican Americans and excluded 809 participants with self-reported history of obesity-related disorders (i.e., heart attack, heart failure, stroke, or peripheral arterial disease) because they could have healthier lifestyles which may lead to reverse causation in cross-sectional studies. In addition, we excluded 245 participants with missing data on the components of the obesity phenotype and 626 participants with missing data on study covariates. The final analytic sample included 5,426 adults with obesity (3,552 women and 1,874 men).

### 2.2. Examination Methods

Participants were asked to fast and refrain from smoking for 12 hours and to avoid physical activity the morning of the examination. Study participation comprised anthropometric assessment, blood draw, medication review, and self-reported sociodemographic and health surveys ascertained via face-to-face interviews by trained, bilingual interviewers. Body weight was measured using the Tanita Body Composition Analyzer (Model TBF-300A). Height was measured to the nearest centimeter and body weight to the nearest 0.1 kg. BMI was calculated as weight in kilograms divided by height in meters squared. After a 5-minute rest period, 3 seated blood pressure measurements and heart rate were obtained with an automatic sphygmomanometer, and the mean of these three readings were used. Blood samples were measured for total serum cholesterol, plasma glucose (fasting and 2-hour postglucose load), and hemoglobin A1c (HbA_1C_) according to standardized protocols. Total serum cholesterol was measured using the cholesterol oxidase enzymatic method. Plasma glucose was measured using the hexokinase enzymatic method (Roche Diagnostics) and hemoglobin A1c using the Tosoh G7 Automated HPLC Analyzer (Tosoh Bioscience Inc). Medication use in the past month was assessed based on self-report.

### 2.3. Study Measures

#### 2.3.1. Obesity Phenotypes

Given that no uniform criteria to define obesity phenotypes exist, we used a previous definition originally developed by researchers from the INTERMAP study [14]. Participants with obesity and absence of cardiometabolic abnormalities (i.e., favorable levels of blood pressure, lipids, and glucose) who met all of the following criteria were classified with the MHO phenotype: systolic blood pressure <120 mmHg, diastolic blood pressure <80 mmHg, and not taking hypertensive medication; fasting triglycerides <200 mg/dL, fasting low-density lipoprotein-cholesterol <160 mg/dL, high-density lipoprotein-cholesterol ≥50 mg/dL in women, fasting high-density lipoprotein-cholesterol ≥40 mg/dL in men, and not taking cholesterol-lowering medication; and fasting plasma glucose <100 mg/and not taking medication for diabetes mellitus. Participants with obesity and ≥1 of the above cardiometabolic abnormalities were classified as the MAO phenotype.

#### 2.3.2. Sociodemographic Characteristics

Information on sociodemographic characteristics (i.e., sex, age, Hispanic/Latino background, education, annual household income, employment, health insurance, nativity, years living in the US, and language preference) was self-reported.

#### 2.3.3. Behavioral Characteristics

Lifestyle factors were self-reported. Mean lifetime cigarette use (measured in packs per year) was calculated as the number of exposure years multiplied by the average number of cigarettes smoked per day and divided by 20. Never and former smokers were assigned a value of zero for this variable. To ascertain mean weekly alcohol intake, participants were asked whether they currently drink alcoholic beverages and the number of servings consumed per week of wine, beer, and liquor/spirits. Total weekly consumption of each beverage was multiplied by the alcohol content (in grams) of the portion size, summed across beverages, and averaged over 7 days according to Dietary Guidelines for Americans [24]. Those reporting never or former alcohol intake were assigned a value of zero for this variable. Dietary data were collected via two 24-hour dietary recalls administered by trained interviewers approximately 6 weeks apart [25]. The Nutrition Data System for Research (NDSR) developed by the Nutrition Coordinating Center at the University of Minnesota was used to conduct diet assessment and nutrient analyses. The 2010 Alternate Healthy Eating Index (AHEI-2010) based on servings per day of 11 components (i.e., vegetables not including potatoes, whole fruit, whole grains, sugar-sweetened beverages and fruit juices, nuts and legumes, red/processed meats, trans fats, long-chain [*n*-3] fats, polyunsaturated fatty acids, sodium, and alcohol) was computed to ascertain diet quality [26]. Physical activity levels (inactive/low vs. medium/high) were categorized as follows according to the 2008 US guidelines for moderate and vigorous physical activity levels [27]: inactive (i.e., no activity beyond baseline activities of daily living); low (i.e., activity beyond baseline but <150 min/week of moderate-intensity physical activity, or <75 minutes/week of vigorous-intensity activity, or a combination of both); medium (i.e., 150–300 minutes/week of moderate-intensity activity, or 75–150 minutes/week of vigorous-intensity physical activity, or a combination of both); and high (i.e., >300 minutes/week of moderate-intensity physical activity, or >150 minutes/week of vigorous activity, or a combination of both).

#### 2.3.4. Body Composition and Clinical Characteristics

Body fat percentage (i.e., body fat divided by weight and multiplied by 100) and lean body mass were measured to the nearest centimeter using bioelectrical impedance analysis (BIA) method [28] with the Tanita Body Composition Analyzer (Model TBF-300A). Waist circumference was recorded to the nearest centimeter at the uppermost lateral border of the right ilium [29]. Fasting blood samples were collected for measurement of insulin levels. A Roche Modular P Chemistry Analyzer was used to analyze serum high-sensitivity C-reactive protein (hsCRP) (Roche Diagnostics Indianapolis, IN). The resting heart rate was recorded three times, and the mean of readings was used.

### 2.4. Statistical Analysis

All analyses were conducted for men and women separately. The age-adjusted proportion of the MHO phenotype was calculated for the overall sample and by sex and Hispanic/Latino background. Descriptive statistics were generated on the distribution of study covariates (i.e., sociodemographic, behavioral, and clinical characteristics) for the overall target population and by obesity phenotypes for women and men. Differences in the distribution of sociodemographic, behavioral, and clinical characteristics by obesity phenotypes in women and men were examined using *χ* ^2^ tests for categorical variables and *F*-tests for continuous variables. Sensitivity analysis was conducted to estimate the MHO proportion using the following definition proposed by Wildman et al. [12]: obesity and presence of 0 or 1 of the following metabolic abnormalities; blood pressure ≥130/85 mmHg or medication use; fasting triglyceride level ≥150 mg/dL; HDL-C level <40 mg/dL in men or <50 mg/dL in women or lipid-lowering medication use; fasting glucose level ≥100 mg/dL or antidiabetic medication use; homeostasis model assessment of insulin resistance (HOMA-IR) >6.54 (i.e., the 90^th^ percentile in HCHS/SOL cohort aged >20 years including participants without obesity); and hsCRP level >8.67 (i.e., the 90^th^ percentile in HCHS/SOL cohort aged >20 years including participants without obesity).

Multivariable Poisson regression models with robust variance were used to examine associations between a 1-standard deviation (SD) increase in each body composition measure (i.e., percentage body fat, waist circumference, and lean body mass as continuous variables) and the prevalence of the MHO phenotype for women and men separately (reference group: MAO phenotype). Prevalence ratios (PRs) and their 95% confidence intervals (CIs) as well as *p* values were computed. Covariates were identified a priori based on a review of related literature and were considered confounders of the associations of interest if they were associated with either the body composition measures in our sample and/or obesity phenotypes. Model 1 adjusted for age only and model 2 (final model) adjusted for sociodemographic (i.e., age, Hispanic/Latino background, education, nativity, language preference, and field center) and behavioral (i.e., lifetime cigarette use, alcohol consumption, diet quality, and physical activity) characteristics. Data management was performed using SAS 9.4 software (SAS Institute, Cary, NC), and all statistical analyses were performed using Stata Statistical Software Release 14 (Stata Corp LP, College Station, TX). All reported values were weighted to account for the disproportionate selection of the sample and to adjust for any bias due to differential nonresponse in the selected sample. Tests of significance were two-sided at a significance level of 0.05.

## 3. Results and Discussion

The target population was mostly females (57.1%), and the mean age was 42.2 years (SD = 14.1). Overall, 38.7% had more than high school education and 42.5% reported an annual household income below $20,000. The mean number of years living in the US was 22.0 (SD = 15.0) (Table 1).

### 3.1. Distribution and Sociodemographic, Behavioral, and Clinical Correlates of Obesity Phenotypes

**Figure 1:**
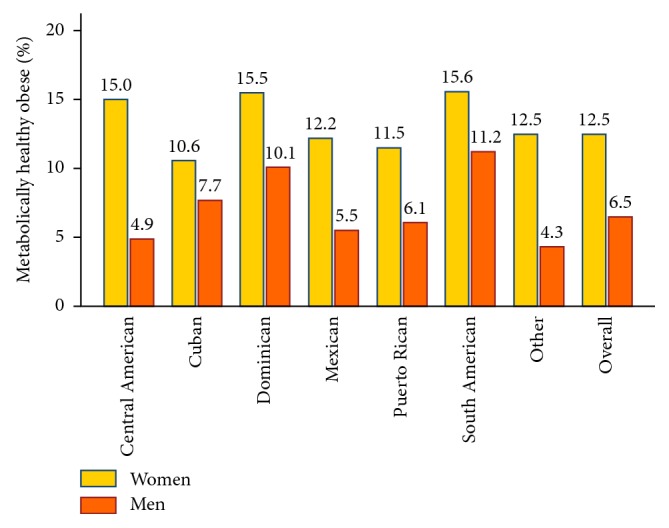
Age-adjusted proportion of metabolically healthy obesity (MHO) phenotype by Hispanic/Latino background for women and men with obesity, HCHS/SOL, 2008–2011.

The age-adjusted proportion of the MHO phenotype was 9.9% (95% CI: 8.8, 11.1) among the overall target population, 12.5% (95% CI: 10.8, 14.2) among women, and 6.5% (95% CI: 5.1, 8.0) among men, and it was the highest in women and men of South American background (Figure 1).

In sensitivity analysis with obesity phenotypes defined according to Wildman et al. [12], the age-adjusted proportion of the MHO phenotype was 36.6% (95% CI: 34.5, 38.7) among the overall target population, and it was higher in women (40.1%; 95% CI: 37.4, 42.9) than in men (32.0%; 95% CI: 29.1, 34.8).

Compared with their counterparts with the MAO phenotype, women and men with the MHO phenotype tended to be younger, have completed high school or more, be born in the US mainland, and preferred English language (all *p* values < 0.05) (Table 1). Women with the MHO phenotype had lived in the US, on average, 3 years less than those with the MAO phenotype (*p*=0.002).

Women with the MHO phenotype reported lower lifetime cigarette use (1.6 vs. 4.0 packs per year; *p* < 0.001) and higher weekly alcohol consumption (2.1 vs. 0.9 grams; *p*=0.024) but had lower diet quality as assessed by the AHEI-2010 score (45.5 vs. 46.8, *p*=0.010), compared with those with the MAO phenotype. Among women, there were no statistically significant differences in weekly physical activity levels between obesity phenotypes. Men with the MHO phenotype reported lower lifetime cigarette use (3.6 vs. 6.0 packs per year; *p*=0.003) and higher weekly physical activity levels (75.0% vs. 63.9% with medium/high levels; *p*=0.038). Among men, there were no statistically significant differences in weekly alcohol consumption and diet quality between obesity phenotypes.

Finally, women with the MHO phenotype had lower mean BMI (34.2 vs. 35.9; *p* < 0.001), lower fasting insulin levels (12.9 vs. 18.1; *p* < 0.001), lower hsCRP levels (5.5 vs. 6.6; *p*=0.015), lower body fat percentage (43.4 vs. 44.7; *p* < 0.001), and lower waist circumference (104.9 vs. 108.6; *p* < 0.001) than those with the MAO phenotype. There were no statistically significant differences in the mean heart rate and lean body mass between obesity phenotypes in women. Men with the MHO phenotype had lower fasting insulin (12.2 vs. 19.5; *p* < 0.001) and heart rate (63.1 vs. 67.0 beats/minute; *p*=0.002) and smaller waist circumference (108.3 vs. 111.4; *p*=0.013), compared with those with the MAO phenotype. There were no statistically significant differences in weekly alcohol consumption, diet quality, BMI, hsCRP, body fat percentage, or lean body mass between obesity phenotypes in men.

In additional secondary analyses (Supplementary Table 1), we examined the multivariable associations between behavioral characteristics and the MHO phenotype for women and men separately (using Poisson regression models). Among women, lifetime cigarette use, diet quality, and physical activity levels were not associated with the MHO phenotype. However, higher weekly alcohol consumption was associated with a higher prevalence of the MHO phenotype among women, regardless of sociodemographic characteristics, other behavioral characteristics, and body composition measures. In adjusted models, there were no associations between any of the behavioral characteristics and the MHO phenotype in adjusted models.

### 3.2. Associations of Body Composition Measures and Metabolically Healthy Obesity Phenotype

Table 2 displays results of multivariable Poisson regression models for the associations between body composition measures (i.e., body fat percentage, waist circumference, and lean body mass) and the MHO phenotype for women and men separately. Among women, higher body fat percentage, higher waist circumference, and higher lean body mass were independently associated with a lower prevalence of the MHO phenotype in models adjusted for age only. These associations persisted after additional adjustment for Hispanic/Latino background, education, nativity, language preference, field center, lifetime cigarette use, alcohol consumption, diet quality, and physical activity. Specifically, in fully adjusted models among women, each increase of 1-SD in body fat percentage (i.e., 5.2%), waist circumference (i.e., 13.6 cm or 5.4 inches), and lean body mass (i.e., 6.6 kg) was, respectively, associated with a 21% (PR: 0.79; 95% CI: 0.69, 0.90), 33% (PR: 0.67; 95% CIs: 0.57, 0.80), and 31% (PR: 0.69; 95% CI: 0.58, 0.81) lower prevalence of the MHO phenotype.

Among men, body fat percentage was not associated with the MHO phenotype in the minimally adjusted model or in the full model. Contrastingly, higher waist circumference was associated with a lower prevalence of the MHO phenotype in the model adjusted for age. This association between waist circumference and the MHO phenotype persisted in the final model. Also, among men, there was no association between lean body mass and the MHO phenotype in the model adjusted for age; but, in the fully adjusted model, there was an association between higher lean body mass and a lower prevalence of the MHO. Specifically, in fully adjusted models among men, each increase of 1-SD in waist circumference (10.8 cm) and lean body mass (i.e., 8.2 kg) was, respectively, associated with a 20% (PR: 0.80; 95% CI: 0.65, 0.99) and 15% (PR: 0.85; 95% CI: 0.74, 0.99) lower prevalence of the MHO phenotype.

## 4. Discussion

In this study, we identified behavioral and clinical characteristics in women and men that differed between obesity phenotypes. Among both women and men, those with the MHO phenotype (vs. MAO) tended to be younger, have higher education, be more acculturated, and have lower lifetime cigarette use, lower fasting insulin levels, and smaller waist circumference. However, there were some characteristics that differed between obesity phenotypes in women but not in men (and vice versa). Women with the MHO phenotype (vs. MAO) had higher weekly alcohol consumption and also had lower diet quality, lower BMI, lower hsCRP levels, and lower body fat percentage. Men with the MHO phenotype (vs. MAO) were more physically active and had lower heart rates. The current study also examined the sex-specific associations between body composition measures and prevalence of the MHO phenotype. Among women, we found that the higher body fat percentage was associated with a lower prevalence of the MHO phenotype, regardless of sociodemographic and behavioral factors, whereas among women and men, higher waist circumference and higher lean body mass were independently associated with a lower prevalence of the MHO phenotype, regardless of sociodemographic and behavioral factors.

The proportion of the MHO phenotype in samples comprising US Hispanics/Latinos has only been investigated in a few studies. In this study, the age-adjusted proportion of Hispanics/Latinos with obesity who met the criteria for the MHO phenotype (i.e., 9.9%) is much lower than the 19% previously reported by Hankinson et al. [14] for a smaller sample comprising diverse adults with obesity (including Mexican Americans) aged 40–59 years from the INTERMAP Study [14]. Although we used the same MHO definition as Hankinson et al. [14], the lower proportion found in our study likely reflects the difference in age range between our samples (i.e., aged 20–74 years in our study vs. 40–59 years in Hankinson et al. [14]). It may also reflect differences in the burden of obesity and CVD risk factors across the racial/ethnic populations and geographic regions that were included in these studies [20, 21]. Interestingly, the 36.6% age-adjusted MHO proportion we found in sensitivity analyses using the Wildman et al. [12] definition was similar to the 33.8% they reported [12] for Mexican Americans with obesity (aged 20–75 years) in NHANES 1999–2004. This suggests that the proportion of the MHO phenotype in Mexican Americans is similar to that of the MHO phenotype in Hispanics/Latinos as a whole.

Consistent with earlier reports [12, 14], the current study showed that persons with the MHO phenotype were younger than those with the MAO phenotype. This finding is also consistent with previous evidence showing that the adverse effects of obesity on cardiometabolic health depend on the duration of obesity [30]. A growing body of research has shown that a majority of those with the MHO phenotype will eventually develop CVD risk factors and, thus, change to having the MAO phenotype [10, 11, 31, 32], suggesting that the MHO phenotype is transitional. Overall, our findings and previous evidence suggest that an important preventive strategy would be to identity individuals with the MHO phenotype and start lifestyle interventions to avoid their transition to the MAO. Additionally, this is the first study, to our knowledge, to examine whether acculturation is related to the obesity phenotype in Hispanic/Latino adults. We found that among women and men, the MHO phenotype was associated with being born in the US mainland and English language preference (two commonly used proxy measures of acculturation). This finding was surprising because there is growing evidence that higher levels of acculturation are associated with adverse profiles of CVD risk factors in Hispanics/Latinos [20, 21]. Our finding may simply reflect that younger Hispanics/Latinos are more likely to be born in the US mainland and prefer to speak English compared with older groups.

In partial support of our hypothesis, in bivariate analyses, more favorable levels of some (but not all) of the behavioral factors examined were associated with the MHO phenotype and some of these associations differed by sex. We found that women and men with the MHO phenotype had lower lifetime cigarette use than those with the MAO phenotype, a finding that is consistent with well-established evidence of associations between cigarette smoking and adverse cardiometabolic health [33]. However, the associations between lifetime cigarette use and obesity phenotypes were not evident in supplementary analyses with adjusted regression models, which suggest the presence of confounders in these bivariate associations (e.g., demographic and other behavioral factors). It was also found that mean levels of weekly alcohol consumption (in grams) were higher in the MHO (vs. MAO) among women but not among men; similar associations were found in adjusted regression models. Some studies have demonstrated benefits of moderate alcohol intake on lipids and glucose levels in women and men [34–36]. It is possible that we were unable to capture the associations of alcohol intake and obesity phenotypes among men if they underreported their alcohol consumption, which would bias our results towards the null. Regarding diet quality, surprisingly, women with MHO phenotypes had significantly lower levels, in average, of diet quality compared with the MAO phenotype. However, these differences in diet quality were relatively small (i.e., about one unit) and there were no differences in diet quality according to obesity phenotypes among men (and no associations between diet quality and obesity phenotypes in adjusted models). In the Dutch Lifelines Study, similarly, there was an association of healthier diet quality and the MHO phenotype in women but not in men [15]. In the INTERMAP study [14], however, no association was observed between diet composition and obesity phenotypes in diverse women and men aged 40 years and older. This discrepancy in findings may be because the INTERMAP study used a diet measure (created by INTERMAP Study investigators) comprising 16 food groups while we used a total diet score (i.e., AHEI-2010). We also found an association between higher physical activity levels and the MHO phenotype in men but not in women, whereas in supplementary analyses, there was no association between physical activity levels and obesity phenotypes in women or men. Some prior studies [12, 16, 17] have reported an association of self-reported physical activity and the MHO phenotype among adults in NHANES, although another study did not find such an association in a diverse sample of adults [14]. Furthermore, in the Dutch Lifelines Study, vigorous physical activity was associated with the MHO phenotype in men but not in women [15]. These inconsistent findings on the associations of behavioral factors with obesity phenotypes by sex may reflect methodological limitations related to using different self-reported measures to assess health behaviors and inconsistent definitions of obesity phenotypes.

As hypothesized, we found that higher body fat percentage was associated with a lower prevalence of the MHO phenotype in women. A previous study [18] similarly found an inverse association between body fat percentage and the MHO phenotype in women, yet men were not included in the sample. However, contrary to our hypothesis, we observed no association between body fat percentage and the MHO in men (although estimates were in the hypothesized direction). In fact, in our analytic sample of adults with obesity, the mean value of body fat percentage (40.4%) is generally considered to be high and the difference in body fat percentage between obesity phenotypes was only about 1%. The lack of association between body fat percentage and the MHO phenotype in men is aligned with previous evidence questioning whether those with the MHO phenotype are actually “healthier” [37]. Although previous evidence on the associations between body fat percentage and CVD risk is scarce, it has been shown that high body fat percentage is more strongly associated with a risk of coronary heart disease (CHD) [38] and mortality [39, 40] than BMI ≥ 30 kg/m^2^.

We also found, as hypothesized, that higher waist circumference was associated with a lower prevalence of the MHO phenotype in women and men. Previous studies have similarly found an inverse association between waist circumference [12, 13] and the MHO phenotype, although, in these available studies, analyses were not stratified by sex. Furthermore, the association we found between higher waist circumference and a lower prevalence of the MHO phenotype is consistent with that of the previous literature which has consistently documented associations between higher abdominal obesity and unfavorable cardiometabolic profiles [41].

We had hypothesized that higher lean body mass would be associated with a higher prevalence of the MHO, given that studies suggest that lean body mass is important for glucose regulation [42] and protective for the development of cardiometabolic abnormalities [43]. But, contrary to our hypothesis, we found that higher lean body mass is associated with a lower prevalence of the MHO among women and men (independently of sociodemographic and behavioral factors). This finding is at odds with the only (to our knowledge) similar previous study [18], which found no association between lean body mass and obesity phenotypes in a sample comprising postmenopausal non-Hispanic white women. A plausible explanation for our contradictory finding is that those with high lean body mass also have high body fat, a combination which has been found to be associated with a 2 times higher incidence of the metabolic syndrome compared with those with low lean body mass and low body fat [43]. As such, future research is needed to better understand the associations of the combined effects of different profiles of muscle mass and fat mass on cardiometabolic abnormalities.

One limitation of this study is the cross-sectional nature of our data, which does not allow for inferences regarding causality. There is some evidence that measures of central adiposity are better predictors of CVD events and mortality than BMI among non-Hispanic whites but considerable debate still exists [44, 45], and prospective studies examining which body composition measure better predicts CVD events and mortality in Hispanics/Latinos are needed. The long-term follow-up in HCHS/SOL will provide an opportunity to examine the incidence of obesity phenotypes in relation to baseline socioeconomic, behavioral, and clinical factors, including body composition measures. Finally, there are no published guidelines on the definitions of obesity phenotypes and definitions vary across studies. However, we used a definition that is comparable with a previous, well-recognized epidemiological study (i.e., INTERMAP) [14] that included data from US adults. Furthermore, the MHO phenotype was defined in the current study using similar but less strict criteria (vs. MAO phenotype) that have been shown to be prospectively associated with a higher risk of type 2 diabetes (but not CVD) [46].

## 5. Conclusions

To our knowledge, this is the first epidemiologic study to examine the age-adjusted distribution of obesity phenotypes and its sociodemographic, behavioral, and clinical correlates in a large, diverse sample of US Hispanic/Latino women and men with obesity. This study demonstrates that only a small proportion of Hispanic/Latino women and men with obesity are metabolically healthy, highlighting the need to reduce the burden of CVD risk factors among Hispanic/Latino adults with obesity through targeted culturally tailored interventions. This study also corroborates previous findings showing an inverse association between waist circumference and the MHO phenotype, which may partially explain the absence of cardiometabolic abnormalities among those with obesity. Our findings highlight the need to use multiple adiposity measures when examining cardiometabolic risk in Hispanic/Latino women and men.

## Figures and Tables

**Table 1:** Characteristics of target population (mean ± standard deviation or *n* (percentage)) according to obesity phenotypes in women and men with obesity, HCHS/SOL, 2008–2011.

	Total *n* = 5426	Women (*n* = 3552)			Men (*n* = 1874)		
		MAO	MHO		MAO	MHO	
		*n* = 3181 (88.1%)	*n* = 371 (11.9%)	*p* Value	*n* = 1743 (92.7%)	*n* = 131 (7.3%)	*p* Value
Age (years)	42.2 ± 14.1	44.6 ± 15.5	35.4 ± 11.3	<**0.001**	41.1 ± 12.2	32.1 ± 9.3	<**0.001**
Hispanic/Latino background, *n* (%)							
Central American	600 (7.4)	346 (7.2)	37 (9.4)	0.058	206 (7.5)	11 (6.1)	0.525
Cuban	728 (19.8)	398 (19.4)	31 (11.7)		286 (22.0)	13 (17.4)	
Dominican	418 (9.2)	264 (10.0)	34 (14.5)		107 (7.1)	13 (12.6)	
Mexican	2312 (38.7)	1377 (38.3)	176 (41.4)		700 (38.7)	59 (38.1)	
Puerto Rican	902 (16.7)	553 (17.2)	48 (13.6)		282 (16.7)	19 (15.1)	
South American	297 (4.0)	162 (3.8)	25 (4.2)		100 (3.9)	10 (6.3)	
Others	169 (4.2)	81 (4.2)	20 (5.2)		62 (4.1)	6 (4.4)	
Education, *n* (%)							
<High school	2101 (33.1)	1336 (37.6)	124 (28.1)	**0.002**	599 (28.9)	42 (24.3)	**0.042**
High school graduate	1369 (28.2)	737 (27.3)	91 (24.1)		512 (30.5)	29 (21.2)	
>High school	1956 (38.7)	1108 (35.1)	156 (47.8)		632 (40.5)	60 (54.5)	
Annual household income, *n* (%)							
≤$20,000	2385 (42.5)	1530 (48.8)	177 (42.7)	0.194	637 (35.4)	41 (31.1)	0.067
$20,001–40,000	1718 (31.4)	929 (29.0)	116 (29.9)		627 (35.2)	46 (26.2)	
$40,001–75,000	716 (13.6)	353 (10.9)	45 (14.1)		287 (15.8)	31 (27.5)	
>$75,000	224 (5.3)	88 (2.6)	13 (5.3)		115 (8.2)	8 (12.0)	
Do not know/refused	383 (7.2)	281 (8.7)	20 (7.9)		77 (5.5)	5 (3.2)	
Employed, *n* (%)	1543 (30.4)	524 (16.2)	81 (20.6)	0.104	874 (48.9)	64 (45.6)	0.567
Health insurance, *n* (%)	2712 (49.1)	1699 (53.5)	179 (47.8)	0.149	783 (44.2)	51 (43.0)	0.847
US born, *n* (%)	1004 (24.0)	516 (19.9)	93 (28.5)	**0.007**	348 (26.8)	47 (42.7)	**0.006**
Time living in US (years)	22.0 ± 15.0	22.4 ± 16.9	19.6 ± 12.7	**0.002**	22.1 ± 13.3	20.3 ± 10.4	0.112
English language preference, *n* (%)	1153 (27.4)	596 (23.7)	103 (36.9)	**0.001**	408 (29.3)	46 (41.3)	**0.033**
Field center, *n* (%)							
Bronx	1247 (28.1)	766 (30.4)	86 (33.5)	0.226	363 (24.0)	32 (32.0)	0.384
Chicago	1477 (17.1)	822 (15.7)	93 (16.5)		523 (18.7)	39 (19.2)	
Miami	1281 (28.6)	725 (28.6)	71 (21.8)		464 (30.1)	21 (25.3)	
San Diego	1421 (26.2)	868 (25.3)	121 (28.2)		393 (27.2)	39 (23.5)	
Behavioral characteristics							
Lifetime cigarette use (pack/yrs)	4.6 ± 12.4	4.0 ± 13.6	1.6 ± 5.5	<**0.001**	6.0 ± 11.9	3.6 ± 6.6	**0.003**
Weekly alcohol consumption (g)	2.6 ± 6.2	0.9 ± 3.3	2.1 ± 6.7	**0.024**	4.7 ± 7.4	3.4 ± 3.9	0.050
Diet quality (AHEI-2010)	47.5 ± 7.4	46.8 ± 8.0	45.5 ± 7.2	**0.010**	48.7 ± 6.6	47.6 ± 5.7	0.082
Weekly PA levels							
Inactive/low	2601 (44.1)	1726 (51.3)	179 (46.8)	0.242	657 (36.1)	39 (25.0)	**0.038**
Medium/high	2825 (55.9)	1455 (48.7)	192 (53.2)		1086 (63.9)	92 (75.0)	
Clinical characteristics							
BMI (kg/m^2^)	35.1 ± 5.1	35.9 ± 5.9	34.2 ± 4.3	<**0.001**	34.5 ± 4.1	33.8 ± 33.6	0.162
Fasting insulin (mU/L)	18.1 ± 13.1	18.1 ± 15.0	12.9 ± 7.0	<**0.001**	19.5 ± 11.5	12.2 ± 7.1	<**0.001**
hsCRP (mg/L)	5.5 ± 6.3	6.6 ± 7.4	5.5 ± 5.8	**0.015**	4.1 ± 4.7	3.6 ± 4.5	0.420
Heart rate (beats/min)	67.2 ± 10.5	67.8 ± 11.3	66.5 ± 9.1	0.083	67.0 ± 9.6	63.1 ± 8.7	**0.002**
Body fat (%)	40.4 ± 7.6	44.7 ± 5.3	43.4 ± 4.4	<**0.001**	34.9 ± 6.1	33.6 ± 6.2	0.101
Waist circumference (cm)	109.4 ± 12.5	108.6 ± 13.8	104.9 ± 11.8	<**0.001**	111.4 ± 10.9	108.3 ± 9.5	**0.013**
Lean body mass (kg)	55.7 ± 11.5	48.5 ± 6.8	48.2 ± 4.6	0.430	65.3 ± 8.3	65.2 ± 6.7	0.962

*Note.* All values were weighted for survey design and nonresponse. *p* Value of comparisons across obesity phenotypes was calculated using chi-square tests for categorical variables and adjusted Wald tests for continuous variables. MAO = metabolically at-risk obesity; MHO = metabolically healthy obesity; BMI = body mass index; hsCRP = high-sensitivity C-reactive protein; PA = physical activity.

**Table 2:** Associations between each 1-standard deviation (SD) increase in body composition measures and metabolically healthy obesity phenotype among Hispanic/Latino women and men with obesity: prevalence ratios (PRs) and 95% CIs.

Body composition	Women		Men	
	*n* = 3552		*n* = 1874	
	SD	PR (95% CI)	SD	PR (95% CI)
Body fat (%)	5.2		6.1	
Model 1		0.80 (0.71, 0.91)^*∗∗*^		0.86 (0.69, 1.07)
Model 2		0.79 (0.69, 0.90)^*∗∗∗*^		0.90 (0.73, 1.10)
Waist circumference (cm)	13.6		10.8	
Model 1		0.67 (0.57, 0.79)^*∗∗∗*^		0.78 (0.63, 0.98)^*∗*^
Model 2		0.67 (0.57, 0.80)^*∗∗∗*^		0.80 (0.65, 0.99)^*∗*^
Lean body mass (kg)	6.6		8.2	
Model 1		0.71 (0.60, 0.83)^*∗∗∗*^		0.86 (0.74, 1.01)
Model 2		0.69 (0.58, 0.81)^*∗∗∗*^		0.85 (0.74, 0.99)^*∗*^

*Note.* At-risk obesity phenotype is the referent group. Standard deviation is weighted and shown for ease of interpretation of results. SD = standard deviation. Model 1: adjusted for age (years). Model 2: adjusted for variables in model 1 plus Hispanic/Latino background, education, nativity, language preference, field center, lifetime cigarette use, alcohol consumption, diet quality, and physical activity. ^*∗*^ *p* < 0.05; ^*∗∗*^ *p* < 0.01; ^*∗∗∗*^ *p* < 0.001.

## Data Availability

The Hispanic Community Health Study/Study of Latinos (HCHS/SOL) is a multicenter epidemiologic study supported by contracts with the National Heart, Lung, and Blood Institute (NHLBI). The dataset used to support the findings of this study is restricted by the governing IRBs that oversee this human subject research in order to protect participants' privacy. Data are available from the data curators at NHLBI through the BIOLINCC website for researchers who meet the criteria for access to confidential data. Interested researchers should visit the BIOLINCC (https://biolincc.nhlbi.nih.gov/home/) to learn how to obtain HCHS/SOL study data. Additionally, the direct link to the Data Request Form for the HCHS/SOL baseline data is https://biolincc.nhlbi.nih.gov/requests/type/hchssol/; however, researchers must first register on the BIOLINCC website for access to this form.

## Supplementary Materials

Supplementary Materials Supplementary Table 1: associations between behavioral characteristics and metabolically healthy obesity phenotype among Hispanic/Latino women and men with obesity—prevalence ratios (PRs) and 95% CIs.
