# Supplementary material for: Correlates of and Body Composition Measures Associated with Metabolically Healthy Obesity Phenotype in Hispanic/Latino Women and Men: The Hispanic Community Health Study/Study of Latinos (HCHS/SOL)
Source: J Obes. 2019 Jan 15;2019:1251456. doi: 10.1155/2019/1251456 (PMC6350597; doi:10.1155/2019/1251456)
Supplement: Supplementary Materials — Supplementary Table 1: associations between behavioral characteristics and metabolically healthy obesity phenotype among Hispanic/Latino women and men with obesity—prevalence ratios (PRs) and 95% CIs. [file 1251456.f1.pdf]

Supplementary Table 1: Associations between behavioral characteristics and metabolically healthy obesity phenotype among Hispanic/Latino women and men with obesity— Prevalence Ratios (PRs) and 95% CIs.

		Women<br>*n* = 3552		Men<br>*n* = 1874
Behavioral Characteristics	*SD*	PR (95% CI)	*SD*	PR (95% CI)
Lifetime Cigarette Use (pack/yrs)	*12.8*		*11.6*	
Model 1		0.82 (0.65, 1.02)		1.06 (0.85, 1.31)
Model 2		0.84 (0.67, 1.05)		1.08 (0.89, 1.31)
Weekly Alcohol Consumption (g)	*4.0*		*7.2*	
Model 1		1.09 (1.03, 1.15)**		0.81 (0.66, 1.01)
Model 2		1.09 (1.02, 1.17)*		0.82 (0.68, 1.00)
Model 3: Model 2 + Body Fat %		1.08 (1.02, 1.15)**		0.90 (0.81, 1.00)
Model 4: Model 2 + Waist Circumference		1.17 (1.12, 1.22)**		0.90 (0.81, 1.00)
Model 5: Model 2 + Body Lean Mass		1.07 (1.01, 1.14)*		0.90 (0.81, 1.00)
Diet quality (AHEI-2010)	*7.9*		*6.6*	
Model 1		1.07 (0.92, 1.24)		1.05 (0.87, 1.26)
Model 2		1.02 (0.84, 1.25)		1.20 (0.93, 1.55)
Weekly PA Levels	*n/a*		*n/a*	
Model 1				
Inactive/Low		Ref.		Ref.
Medium/High		-0.03 (-0.29, 0.23)		0.26 (-0.21, 0.74)
Model 2				
Inactive/Low		Ref.		Ref.
Medium/High		-0.02 (-0.27, 0.23)		0.28 (-0.20, 0.77)

Note: At-risk obese phenotype is the referent group. Standard deviation is weighted and shown for ease of interpretation of results.

Abbreviations: SD = Standard Deviation; NA = Does not apply.

Model 1: Adjusted for age (years).

Model 2: Adjusted for variables in model 1 plus Hispanic/Latino background, education, nativity, language preference, field center, and other behavioral characteristics.

**p*<0.05; ***p*<0.01; ****p*<0.001
